# Supplementary material for: Mechanisms governing the pioneering and redistribution capabilities of the non-classical pioneer PU.1
Source: Nat Commun. 2020 Jan 21;11:402. doi: 10.1038/s41467-019-13960-2 (PMC6972792; doi:10.1038/s41467-019-13960-2)
Supplement: Supplementary file 7 — Source data [file 41467_2019_13960_MOESM7_ESM.zip › Source_Data/Figure6/Figure6B_MotifScanOutput/ETS-specific/homerResults/motif2.similar.html]

motif2

## Information for motif2

C
A
T
G
C
A
T
G
G
A
C
T
C
A
G
T
A
T
C
G
G
A
C
T
C
T
A
G
A
T
C
G
A
G
C
T
G
A
C
T
C
G
A
T
  
Reverse Opposite:  

G
C
T
A
C
T
G
A
T
C
G
A
T
A
G
C
G
A
T
C
C
T
G
A
T
A
G
C
G
T
C
A
C
T
G
A
G
T
A
C
G
T
A
C
  

|  |  |
| --- | --- |
| p-value: | 1e-725 |
| log p-value: | -1.670e+03 |
| Information Content per bp: | 1.573 |
| Number of Target Sequences with motif | 2936.0 |
| Percentage of Target Sequences with motif | 38.35% |
| Number of Background Sequences with motif | 4060.3 |
| Percentage of Background Sequences with motif | 10.06% |
| Average Position of motif in Targets | 100.5 +/- 47.3bp |
| Average Position of motif in Background | 97.9 +/- 60.7bp |
| Strand Bias (log2 ratio + to - strand density) | 0.1 |
| Multiplicity (# of sites on avg that occur together) | 1.26 |
| Motif File: | file (matrix) reverse opposite |

### Similar de novo motifs found

|  |  |  |  |  |  |  |  |
| --- | --- | --- | --- | --- | --- | --- | --- |
| Rank | Match Score | Redundant Motif | P-value | log P-value | % of Targets | % of Background | Motif file |
| 1 | 0.988 | G A C T C G A T T C A G G A C T T C A G T A C G A G C T G A C T C G A T | 1e-706 | -1625.870216 | 37.57% | 9.86% | motif file (matrix) |
| 2 | 0.982 | G A C T C G A T C A T G G C A T C T A G A C T G A C G T G A C T C G T A G C T A | 1e-679 | -1564.281770 | 33.98% | 8.19% | motif file (matrix) |
| 3 | 0.988 | A C T G A C G T G A C T C A G T A C T G G C A T A T C G A C T G A G C T A G C T C G A T A G C T | 1e-673 | -1550.668802 | 36.76% | 9.84% | motif file (matrix) |
| 4 | 0.965 | G C T A C T G A T C G A A G T C A G T C C G T A A T G C G C T A | 1e-648 | -1493.046620 | 42.69% | 13.93% | motif file (matrix) |
| 5 | 0.962 | A C G T A C T G A G C T A C T G A C T G A G C T G A C T | 1e-561 | -1292.224659 | 38.08% | 12.38% | motif file (matrix) |
| 6 | 0.963 | T C G A T C G A G T A C A G T C C G T A A T G C G T C A C T A G T G A C G T C A T G C A C G T A T A C G | 1e-493 | -1136.136851 | 27.88% | 7.32% | motif file (matrix) |
| 7 | 0.924 | T G C A T C G A G T C A T C G A T C G A T A G C A T G C C T G A T A G C G T C A T C A G G C T A A C T G | 1e-481 | -1109.677686 | 29.68% | 8.51% | motif file (matrix) |
| 8 | 0.918 | T G C A G T A C T G A C T C A G T C A G G T C A C G T A T C G A A G T C A G T C G T C A T A G C G C T A C T A G | 1e-456 | -1050.011344 | 22.63% | 5.05% | motif file (matrix) |
| 9 | 0.804 | A T C G G C A T C G T A C G T A A G T C G A T C C T A G A G T C G T C A | 1e-422 | -973.973938 | 45.94% | 20.94% | motif file (matrix) |
| 10 | 0.805 | G C A T G C T A C T G A A T G C G A T C C T A G A G T C | 1e-404 | -931.419813 | 44.13% | 19.98% | motif file (matrix) |
| 11 | 0.847 | T G C A A C T G A T C G G T C A G A C T C T A G G C A T A C T G A T C G A G C T | 1e-398 | -918.670173 | 29.03% | 9.49% | motif file (matrix) |
| 12 | 0.872 | G A C T C T A G C G A T C T A G T C A G A T C G G A C T C A T G T C A G | 1e-393 | -906.555810 | 46.60% | 22.22% | motif file (matrix) |
| 13 | 0.812 | A C G T A C T G C G A T T A G C A C T G A C G T A C G T | 1e-369 | -850.800943 | 26.87% | 8.69% | motif file (matrix) |
| 14 | 0.888 | C G A T A G C T A C T G A C G T C T A G A C T G A C G T A G C T | 1e-364 | -838.391747 | 25.42% | 7.91% | motif file (matrix) |
| 15 | 0.828 | A G C T A C G T A C T G A G C T A C T G A C T G A T C G | 1e-328 | -756.641281 | 47.15% | 24.54% | motif file (matrix) |
| 16 | 0.813 | A G C T G T C A T G C A C T G A A T G C T G A C C T A G T G A C G C T A T C A G | 1e-304 | -700.342451 | 24.17% | 8.24% | motif file (matrix) |
| 17 | 0.769 | A C T G A C T G C G T A A C G T A C T G A C G T A C T G A C T G | 1e-303 | -698.651276 | 12.11% | 1.94% | motif file (matrix) |
| 18 | 0.862 | C A T G C G A T C T G A C T A G T C A G G T C A C A G T A C T G C G A T A C T G A T C G A C G T A C G T C G T A | 1e-243 | -561.467592 | 11.63% | 2.34% | motif file (matrix) |
| 19 | 0.871 | C A T G A T C G A C G T A G T C G A C T A G T C A C G T A T C G C G A T C T A G A C T G A C G T A G C T C G T A | 1e-228 | -525.593991 | 25.87% | 11.05% | motif file (matrix) |
| 20 | 0.795 | A C T G C G A T A C T G A T C G A G C T A C G T A C G T | 1e-225 | -518.514551 | 34.25% | 17.36% | motif file (matrix) |
| 21 | 0.786 | C A G T A C G T A G T C G C A T A C G T C G A T A T C G G C T A C G A T A T C G G C A T A C T G A C T G A C G T | 1e-153 | -353.467772 | 7.39% | 1.47% | motif file (matrix) |
| 22 | 0.770 | A G C T A C G T G C T A T C G A A T G C G T A C C G T A A T G C G T C A A G C T A T G C A G T C | 1e-86 | -198.067093 | 3.29% | 0.48% | motif file (matrix) |
| 23 | 0.802 | C T A G A G T C A G T C A G T C C G T A A G T C C G T A | 1e-83 | -191.437717 | 14.66% | 7.42% | motif file (matrix) |
| 24 | 0.792 | G C A T C A G T A T C G A G T C A G T C C G T A A G T C C T G A | 1e-76 | -176.382612 | 17.94% | 10.16% | motif file (matrix) |
| 25 | 0.774 | G A C T G A C T T G C A T C G A G T A C G A T C C G T A A T G C G A C T C T G A C T A G | 1e-76 | -175.170048 | 6.88% | 2.40% | motif file (matrix) |
| 26 | 0.791 | A C G T A T C G G A C T A C T G A T C G A G C T C T A G T C G A T C G A | 1e-60 | -140.271722 | 9.31% | 4.38% | motif file (matrix) |
| 27 | 0.765 | A G T C C G T A A T C G A G T C A G T C C G T A A G T C G T C A | 1e-34 | -78.995826 | 9.34% | 5.46% | motif file (matrix) |
